# Cross-validation of the RT-QuIC assay for the antemortem detection of chronic wasting disease in elk

**DOI:** 10.1080/19336896.2020.1716657

**Published:** 2020-01-23

**Authors:** N.J. Haley, R. Donner, D.M. Henderson, J. Tennant, E.A. Hoover, M. Manca, B. Caughey, N. Kondru, S. Manne, A. Kanthasamay, S. Hannaoui, S.C. Chang, S. Gilch, S. Smiley, G. Mitchell, A.D. Lehmkuhl, B.V. Thomsen

**Affiliations:** a Department of Microbiology and Immunology, College of Graduate Studies, Midwestern University, Glendale, AZ, USA; b Prion Research Center, Department of Microbiology, Immunology, and Pathology, College of Veterinary Medicine and Biomedical Sciences, Colorado State University, Fort Collins, CO, USA; c TSE/Prion Biochemistry Section, Laboratory of Persistent Viral Diseases, Rocky Mountain Laboratories, National Institute of Allergy and Infectious Diseases, Hamilton, MT, USA; d Department of Biomedical Sciences, College of Veterinary Medicine, Iowa State University, Ames, IA, USA; e Calgary Prion Research Unit, University of Calgary, Calgary, Alberta, Canada; f Hotchkiss Brain Institute, University of Calgary, Calgary, Alberta, Canada; g Department of Comparative Biology and Experimental Medicine, University of Calgary, Calgary, Alberta, Canada; h National and OIE Reference Laboratory for Scrapie and CWD, Canadian Food Inspection, Agency, Ottawa Laboratory-Fallowfield, Ottawa, Ontario, Canada; i United States Department of Agriculture, APHIS, VS, National Veterinary Services Laboratories, Ames, IA, USA; j United States Department of Agriculture, APHIS, VS, Center for Veterinary Biologics, Ames, IA, USA

**Keywords:** Prion, amplification, RT-QuIC, cross-validation, RAMALT

## Abstract

Chronic wasting disease is a progressively fatal, horizontally transmissible prion disease affecting several members of the cervid species. Conventional diagnosis relies on ELISA or IHC evaluation using tissues collected post-mortem; however, recent research has focused on newly developed amplification techniques using samples collected antemortem. The present study sought to cross-validate the real-time quaking-induced conversion assay (RT-QuIC) evaluation of rectal biopsies collected from an elk herd with endemic CWD, assessing both binary positive/negative test results as well as relative rates of amplification between laboratories. We found that results were correlative in both categories across all laboratories performing RT-QuIC, as well as to conventional IHC performed at a national reference laboratory. A significantly higher number of positive samples were identified using RT-QuIC, with results seemingly unhindered by low follicle counts. These findings support the continued development and implementation of amplification assays in the diagnosis of prion diseases of veterinary importance, targeting not just antemortem sampling strategies, but post-mortem testing approaches as well.

## Introduction

Our ability to detect prion infections in humans and animals has evolved rather rapidly over the past two decades, though it has not quite approached the implementation into diagnostic protocols seen with the advent of nucleic acid amplification techniques in the late twentiethth century [1,2]. New prion-specific amplification techniques, including serial protein misfolding cyclic amplification (sPMCA [3]) and real-time quaking-induced conversion (RT-QuIC [4]), have provided for extremely sensitive detection of prions in experimental and clinical samples. Like sensitivity, the specificity of amplification assays themselves varies across techniques and laboratories, though both sensitivity and specificity are generally considered equitable to that of nucleic acid PCR provided adequate and appropriate controls are included [2].

Although cross-validational testing using a standardized protocol has shown inter-lab repeatability for RT-QuIC analysis of human clinical specimens [5], little such testing has been applied towards animal diseases – a factor critical for its potential use in a veterinary diagnostic setting. The RT-QuIC assay was only recently added to the Centres for Disease Control’s diagnostic criteria for Creutzfeldt–Jakob Disease (CJD) based on extensive testing of human CSF and nasal brush collections across multiple laboratories [5–11]. Despite a storied history of testing applications for animal prion diseases, specifically scrapie of sheep and chronic wasting disease (CWD) of cervids [12–30], neither the RT-QuIC assay nor sPMCA has been adopted for routine diagnostic use in the veterinary field, in part because no cross-validational studies have been conducted in animal species.

Amplification assays, in particular, the RT-QuIC assay due to its reliance on recombinant protein substrate and quantitative nature, offer several advantages over conventional testing for both human and animal prion diseases [2]. Apart from their reported improvements in sensitivity, they can be used to evaluate bodily fluids and other samples otherwise untestable by conventional assays like enzyme-linked immunosorbent assay (ELISA) or immunohistochemistry (IHC) [7,9,11,19–22,31-41]; notably, however, the sensitivity of these assays on body fluids and excreta still does not approach the sensitivity achievable using more conventional tissues – often only accessible post-mortem. Amplification assays may also make use of much smaller tissue biopsies than those required for IHC, expediting the sample collection process and potentially increasing the number of longitudinal samples available from a subject [42]. These samples may additionally be positive independent of specific markers (e.g. follicle count or *PRNP* genotype) which may affect conventional diagnosis [14,18,43]. Importantly, amplification assays, like ELISA, can be streamlined and scaled to allow for rapid and automated testing of large numbers of samples, with real-time readouts independent of the need for specialized pathology training.

This manuscript presents a cross-validational study of the application of RT-QuIC testing to recto-anal mucosa associated lymphoid tissue (RAMALT) biopsies collected antemortem from 471 individual Rocky Mountain elk (*Cervus elaphus canadensis*) in a controlled area, with endemic CWD approaching 33% prevalence over the course of a two-year study [14,43]. Testing by RT-QuIC was performed by five different laboratories across the United States and Canada, with results compared between laboratories and to IHC of RAMALT, as well as conventional post-mortem testing of the brainstem and retropharyngeal lymph nodes by IHC, when available. The samples were initially tested by RT-QuIC, blindly, at the primary laboratory (‘Laboratory A’) as part of a larger study, with IHC also performed blindly and concurrently by a national regulatory laboratory; aspects of this study are reported elsewhere [14,43]. Samples were subsequently distributed to and assayed concurrently by the four remaining laboratories (‘Laboratories B through E’) at times ranging between 3 and 15 months post-collection. We hypothesized that test results would correlate not just across those labs performing RT-QuIC, but also across methodologies (e.g. RT-QuIC and IHC). We also hypothesized that the ranked relative rates of amplification in the RT-QuIC assay would strongly correlate across laboratories and would correlate with the number of positive follicles identified in IHC.

We found that, indeed, positive and negative test results correlated very well across laboratories, with RT-QuIC providing a significant increase in test sensitivity over conventional IHC in the antemortem detection of CWD affected elk. Correlation of RT-QuIC testing was highest among laboratories performing the testing concurrently (i.e. Laboratories B through E). Ranked rates of amplification in positive samples were also moderately correlative across laboratories performing RT-QuIC, and to the percentage of positive follicles identified using IHC. Collectively, these findings are an important step towards the further application of prion amplification assays in a conventional diagnostic setting for both cervids as well as humans.

## Materials and methods

### Ethics statement

The animals providing the samples in this study were handled humanely in accordance with Midwestern University’s Animal Care and Use Committee, approval #2814.

### Study population and design

The study population was made up of 471 adult elk from a private herd, with sampling conducted annually over 2 years in a modern handling facility [14]. During the yearly inventory in late winter of 2016 and 2017, samples were collected with physical restraint and submitted for primary testing by both conventional IHC at the United States Department of Agriculture’s National Veterinary Services Laboratories and RT-QuIC at Colorado State University’s Prion Research Centre (Laboratory A). A total of 702 samples were collected and analysed, with 387 collected in the first year and 315 collected in year two. A lengthy description of sampling techniques and handling procedures are described elsewhere [43]. In the spring of 2017 (15 months after initial collection of year one biopsies and 3 months after collection of year two biopsies), frozen sample homogenates were distributed to several CWD-focused laboratories across the United States and Canada with an expressed interest in cross validating the original results (Laboratories B through E). Storage conditions of −20°C to −80°C were maintained as best as possible during both archiving and distribution of the samples between laboratories. A common set of controls, reaction conditions, and evaluation metrics, all described below, were distributed along with the samples for consistent application of methodology. Amplification log files were collected, evaluated and used to confirm results from the originating laboratory.

### Immunohistochemistry testing

Rectal biopsies and post-mortem samples were evaluated for PrP^CWD^ immunostaining as described previously, blindly and without information on the index test (RT-QuIC) results [14]. Briefly, immunohistochemical staining for PrP^CWD^ was performed using the primary antibody Anti-prion 99 (Ventana Medical Systems, Tucson, AZ) and then counter-stained with haematoxylin. Positive and negative controls were included in each analysis. Biopsies were considered positive if at least one follicle exhibited PrP^CWD^-specific staining. Follicle counts, including total number of follicles and the number of positively staining follicles, were included in the analysis. It is important to note that, in experimental settings, RAMALT follicle counts of five or fewer have been associated with poorer CWD testing sensitivity [44,45].

### RT-QuIC testing

Rectal biopsy subsections were prepared as 10% homogenates in phosphate-buffered saline (PBS) and analysed for PrP^CWD^ conversion activity consistently, independently, and blindly across each of the five collaborating laboratories. RT-QuIC assays were performed using a truncated form of the recombinant Syrian hamster prion protein (SHrPrP, residues 90–231), expressed in pET41b and purified as previously described [4,14]. Rectal biopsy homogenates were first diluted from the archived sample 1:100 in RT-QuIC dilution buffer (0.05% sodium dodecyl sulphate in PBS). Five microlitres of this 10^−2^ dilution were then added to 95 µl of RT-QuIC reaction buffer, consisting of 50 mM NaPO4, 350 mM NaCl, 1.0 mM EDTA, 10mM thioflavin T (ThT), and 0.1 mg/ml truncated Syrian hamster rPrP^C^, to yield a final volume of 100 µl. Each test sample was repeated in triplicate on a single plate. Positive controls, consisting of 5 µl of a 10^−3^ dilution of pooled CWD-positive brains from six experimentally infected white-tailed deer (cervid brain pool 6, CBP6) spiked into 95 µl of RT-QuIC reaction buffer, were included in triplicate in each experiment. This control has well-characterized rates of amplification in RT-QuIC and has been titrated in transgenic Tg[CerPrP] mice [14,17,18,23,46]. Negative controls consisted of three RAMALT biopsies from known CWD negative deer, and were also repeated in triplicate. Reactions were prepared in black 96-well, optical-bottom plates, which were then sealed and incubated in a BMG Labtech PolarstarTM fluorimeter at 42⁰C for 24 h (96 cycles, 15 min each) with intermittent shaking; specifically, 1 min shakes (700 rpm, double orbital pattern) interrupted by 1 min rest periods. ThT fluorescence measurements (450 nm excitation and 480 nm emission) were taken every 15 min with the gain set at 1800. The relative fluorescence units (RFU) for each triplicate sample were progressively monitored against time with orbital averaging and 20 flashes/well at the 4 mm setting.

Criteria for identification of positive samples were determined *a priori*. A replicate well was considered positive when the relative fluorescence crossed a pre-defined positive threshold, calculated as ten standard deviations above the mean fluorescence of all sample wells, including positive controls, negative controls, and test samples, from cycles 2–8. Positive samples were those which crossed the threshold in at least two of three replicates. Amplification rates for positive samples were defined as the inverse of the time for a sample to cross the predefined threshold.

### Data assembly and statistical analysis

For RT-QuIC testing, data provided from each laboratory included information on samples with positive and negative results, as well as relative rates of amplification compared to the standardized positive controls. These findings were confirmed by secondary evaluation of the amplification log files, also provided. All samples which were positive in any of the participating laboratories were then ranked based on their rates of amplification relative to a standard positive control, with samples demonstrating rapid rates of amplification ranked highest, and those with low or no amplification ranked lowest. Ties were considered where amplification rates were identical (e.g. those with no amplification).

For IHC testing, data collected included both positive and negative staining results, as well as the number of positive follicles and the total number of follicles observed in a tissue section. For statistical evaluation, all samples which were otherwise found positive by RT-QuIC were ranked based on the percentage of positive follicles observed, with samples having the highest percentage of positive follicles ranked highest. In cases where RT-QuIC testing was positive with no positive follicles observed by IHC, samples were continuously ranked such that those with a larger number of negative follicles were ranked lowest, while those with smaller numbers of negative follicles (or no follicles present) were ranked subsequent to those samples with positive follicles. Ties were considered where the percentage of positive follicles, or the number of negative follicles in the case of IHC negative samples, were identical. The outcome was that samples with a high percentage of positive follicles were ranked highest, while IHC negative samples with the largest number of negative follicles were ranked lowest.

For the initial comparisons across laboratories, a Fleiss’s Kappa analysis, which assesses the reliability of multiple raters of categorical data sets, was used to examine correlations between positive and negative results across (1) all laboratories performing IHC and RT-QuIC, (2) those performing RT-QuIC only, and (3) those performing RT-QuIC concurrently (e.g. Laboratories B through E). A secondary comparison to post-mortem data was also conducted using a Fleiss’s Kappa analysis. The determination is a standard Kappa value of agreement, ranging from zero (no agreement) to one (full agreement) [47,48]. A Kendall’s W analysis, which examines the ranked agreement among multiple raters, was used to evaluate ranked positive samples across all laboratories, between laboratories performing RT-QuIC only, and those conducting RT-QuIC concurrently [49]. The determination in this case is a W value of agreement, ranging from zero (no agreement) to one (full agreement). Interpretation of both K and W values is somewhat arbitrary, with values between 0.41 and 0.60 considered in moderate agreement, 0.61–0.80 considered substantial agreement, and 0.81–1.00 near perfect agreement [47,48]. In both cases, correlation analyses were generated using the Real Statistics Resource Pack software (Release 6.2) [50].

A conventional chi-square test of association was used to evaluate the level of agreement between RT-QuIC, IHC, and post-mortem testing, while a standard t-test was used to compare the mean follicle counts for RT-QuIC positive, IHC positive and RT-QuIC positive, IHC negative samples.

## Results

### Statement of transparency on RT-QuIC testing

In each of the laboratories, a number of amplification plates were discarded as a result of either failed amplification of positive controls (n = 12), amplification observed in one or more negative control replicates over the course of a 24-h study (n = 7), or equipment malfunction (n = 1). Of the 130 total plates run across five laboratories, 20 plates (15%) were discarded with testing repeated. These discarded experiments are presented for the sake of transparency and are summarized in Table 1.

**Table 1.** Summary of testing results from real-time quaking-induced conversion (RT-QuIC) and immunohistochemistry (IHC) on rectal biopsies from elk. Biopsies were collected from ranched elk in a CWD endemic area and tested by RT-QuIC in multiple laboratories in the United States and Canada, with additional testing performed by IHC at the United States Department of Agriculture’s National Veterinary Services Laboratory (NVSL). Number of discarded experimental plates in each laboratory is shown. Correlation between test results was done using a Fleiss’s Kappa analysis, measuring the agreement between positive and negative results, as well as Kendall’s W, which measures the ranked agreement between multiple raters. Key: ^a^ plates discarded when positive controls failed to amplify, ^b^ plates discarded as a result of non-specific amplification in negative controls, ^c^ plates discarded as a result of equipment malfunction.

		RT-QuIC Laboratory													
		A		B		C		D		E					
	Discarded Plates	3^a^, 1^b^ 1^c^		2^a^		1^a^, 5^b^		3^a^, 1^b^		3^a^		NVSL (IHC)		Cumulative Ante- and Post-mortem	
	Assay Results	Pos.	Neg.	Pos.	Neg.	Pos.	Neg.	Pos.	Neg.	Pos.	Neg.	Pos.	Neg.	Pos.	Neg.
Year 1	# of samples	63	324	58	329	58	329	58	329	57	330	33	354	72	315
	Fleiss’s Kappa	0.84													
		0.92													
				0.95											
	Kendal’s W	0.47													
		0.49													
				0.61											
Year 2	# of samples	67	248	67	248	63	252	68	247	67	248	54	261	100	215
	Fleiss’s Kappa	0.85													
		0.89													
				0.90											
	Kendal’s W	0.59													
		0.62													
				0.65											

### Inter-laboratory agreement of CWD positive and negative status

When simply considering the positive and negative results across laboratories performing either RT-QuIC or IHC, the correlation as indicated by Fleiss’s Kappa was very high in samples collected in both years one (0.84) and two (0.85) of the study (Table 1). Agreement improved when only considering laboratories performing RT-QuIC (0.92 and 0.89 in years one and two, respectively); agreement was higher still in those laboratories evaluating samples concurrently (0.95 and 0.90 in years one and two, respectively). When comparisons were made across samples for which post-mortem data were available (Table 2), agreement was also high when considering test results of laboratories performing RT-QuIC and IHC (Year one: 0.88, year two: 0.76) and those performing RT-QuIC alone (Year one: 0.87, year two: 0.75). Agreement was lower when solely considering antemortem IHC and post-mortem IHC results (Year one: 0.77, year two: 0.53).

**Table 2.** Summary of testing results from real-time quaking-induced conversion (RT-QuIC) and immunohistochemistry (IHC) on rectal biopsies from elk with available post-mortem test results. For comparisons between antemortem RT-QuIC ± antemortem IHC and post-mortem IHC where available, a Fleiss’ Kappa analysis was used to measure the agreement between positive and negative results. For comparisons between antemortem IHC and post-mortem IHC where available, a Cohen’s Kappa analysis was used to measure agreement.

		RT-QuIC Laboratory													
		A		B		C		D		E		NVSL Antemortem IHC		Post-mortem IHC	
	Assay Results	Pos.	Neg.	Pos.	Neg.	Pos.	Neg.	Pos.	Neg.	Pos.	Neg.	Pos.	Neg.	Pos.	Neg.
Year 1	# of samples	18	38	19	37	18	38	19	37	18	38	18	38	24	32
	Fleiss’s Kappa		0.88												
		0.87													
												0.77			
Year 2	# of samples	32	91	32	91	29	94	32	91	33	90	33	90	62	61
	Fleiss’s Kappa		0.76												
		0.75													
												0.53			

### Inter-laboratory agreement of positive samples ranked by amplification rates and percentage of positive follicles

The ranked agreement across both RT-QuIC and IHC methodologies ranged from moderate to substantial as indicated by Kendall’s W analysis (Table 1). Again, when considering both RT-QuIC and IHC testing methodologies, ranked agreement was lowest (0.47 and 0.59 in years one and two, respectively). A modest improvement in agreement was observed when considering RT-QuIC alone (0.49 and 0.62 in years one and two, respectively). Agreement was highest in those labs performing RT-QuIC concurrently – 0.61 and 0.65 in years one and two, respectively. In all cases, agreements were again considered significant (p < 0.001).

### Assessment of RT-QuIC and IHC in the antemortem detection of CWD infection in RAMALT

The argument of whether an RT-QuIC positive, IHC negative RAMALT sample is indeed positive is made elsewhere [14,43] and is in general supported by the strong correlation between RT-QuIC results across all laboratories performing the assay. Considering that elk with RT-QuIC positive, IHC negative biopsies had three specific fates in the associated studies: 1) to be harvested within the year and found positive post-mortem in the retropharyngeal lymph nodes (n = 1/66, 1.5%) or both obex and retropharyngeal lymph nodes (n = 7/66, 11%); 2) to return the following year and present as IHC positive antemortem and post-mortem, in both obex and retropharyngeal lymph nodes (n = 14/66, 21%); or 3) to perish untested in the field at a rate six times that of their test-negative cohorts (n = 44/66, 67%), it’s plausible to assume that these animals were all in fact infected with CWD. Said more precisely, no animals found to be RT-QuIC positive antemortem were later found to be IHC negative post-mortem.

While RT-QuIC proved quite adept at identifying CWD positive elk, including those that were IHC negative, it remained imperfect. Of the 77 unique animals identified antemortem as IHC positive across both years of sampling, 14 samples were RT-QuIC negative in one or more of the collaborating laboratories in both study years. Perhaps more importantly, a single sample was found to be IHC positive by antemortem IHC while not having at least one corresponding RT-QuIC result at any of the participating laboratories. In contrast, nearly twice as many positives were found by RT-QuIC – 142 unique animals in total (p < 0.001) across all collaborating laboratories. It is important to note that it is possible some additional number of IHC positives may have been discovered had a multi-lab, standardized IHC study been performed in tandem with the present study. An assessment must then be made as to how effective RT-QuIC is in identifying CWD-positive cervids antemortem and what improvement there is, if any, over antemortem IHC.

Considering animals identified as CWD-positive either antemortem or post-mortem by RT-QuIC, IHC, or both, there were 172 total unique animals found infected across the two-year study period. Conservatively assuming that all infected animals were correctly identified would place the sensitivity of blinded, antemortem IHC of RAMALT tissue at 45% (77/172). The sensitivity of blinded, antemortem RT-QuIC testing combining data from all laboratories was significantly higher, at 83% (142/172, p < 0.001). Both estimates should be considered preliminary, as it is likely that additional CWD positive animals perished in the field and were not tested. Although blinded testing in each individual lab performing RT-QuIC still offered significantly higher sensitivity than IHC (p < 0.01 in all cases), an individual laboratory’s sensitivity was moderately, though significantly reduced (p < 0.05 in all cases) compared to combined RT-QuIC data from all laboratories, and ranged from 65% to 70%. Randomly pairing any of the two laboratories data together resulted in sensitivities lower than, yet statistically indistinguishable from results achieved by all RT-QuIC laboratories combined (p > 0.05 in all pairings).

### Mean follicle counts of IHC positive and negative samples that were positive by RT-QuIC

Previous studies have shown that low follicle counts may affect the sensitivity of IHC for the antemortem detection of CWD in cervids [44,45]. We compared the total number of reported follicles in two groups of samples: those that were RT-QuIC positive and IHC positive, and those that were RT-QuIC positive and IHC negative, to see if low follicle counts might explain the discrepancies between reporting for the two assays. We found that follicle counts were indeed different between these two groups, with RT-QuIC positive/IHC positive samples having a mean follicle count of 33.3 (s.d. ± 30.4), while RT-QuIC positive/IHC negative samples had a mean follicle count of 23.2 (s.d. ± 26.0, p = 0.036). Of the 65 samples which were RT-QuIC positive and IHC negative, fifteen had follicle counts of five or fewer (24.6%), and were considered non-diagnostic. Apart from those fifteen samples, the average follicle count was 30.0 ± 26.7.

## Discussion

While substantial progress has been made in the development of amplification tests for prion diseases in both humans and animals, the clinical adaptation of these tests has so far only included the acceptance of RT-QuIC as a supportive assay for CJD diagnosis by the human medical field [8]. While there is an intrinsic value for the development and application of tests for comparatively rare human diseases, there is an increasingly significant need for accurately detecting prion infections in animals, in particular chronic wasting disease of cervids, which is horizontally transmissible and expanding at a rapid pace [51].

There are currently an extensive list of experimental reports on the ability of prion amplification tests like the RT-QuIC assay to detect CWD prions in a range of clinical samples, including CSF [11,19,34,52], saliva [21,22], urine [21,40,53], blood [31,46,54–56], faeces [23,30,57,58], as well as tissues collected both post-mortem and antemortem [13,17,18,20,59–61]. This study attempts to consolidate the efforts of several laboratories across North America in an effort to show the repeatability and improved sensitivity of the RT-QuIC assay in the antemortem diagnosis of CWD in elk using rectal biopsies. It stands to reason that these advancements in antemortem testing may at least partially transfer to the post-mortem diagnosis of CWD in all susceptible cervids as well. Verifying the true status of RT-QuIC positive, IHC negative tissues collected post-mortem, however, is much more challenging – requiring time-, labour-, and animal-intensive bioassay experiments [59]. These challenges make longitudinal antemortem sampling studies with natural animal model systems much more appealing and informative.

Using two categories of correlation, infection status and ranked rates of amplification, we report that the RT-QuIC assay has a considerable level of correlation both across laboratories, as well as with conventional immunohistochemistry. Correlation was highest in both categories across laboratories performing RT-QuIC, although the ranking of IHC results for the Kendall’s W analysis was admittedly and inherently challenging, resulting in a somewhat diminished correlation between RT-QuIC ranking and IHC ranking. Generally, RT-QuIC correlation was highest among laboratories B through E, which evaluated all samples concurrently and some months after the primary laboratory examined them.

While previous reports of the amplification of prions in human CSF have suggested there was no obvious decay of sample integrity after multiple freeze-thaws [7,11], it cannot be ruled out as a factor in the discrepancies reported here. There is anecdotal evidence in the present study of a combined effect of sample degradation and an apparent ‘Goldilocks effect,’ – wherein those samples with high levels of prions may not amplify well soon after collection, while those with low levels may not amplify well after lengthy periods of storage (data not shown). Other important factors that are likely to help explain the small variation in test results include, most importantly, the quality of recombinant protein produced and used in the assays [2]. Technical expertise and errors likely represent an additional factor. The development of standardized kits and appropriate training would likely decrease the influence of these factors on testing repeatability in the future.

Across both years of study, RT-QuIC performed significantly better than IHC in detecting CWD positive animals. This was partly associated with a lower follicle count in RT-QuIC positive/IHC negative samples compared to those that were positive by both assays; however, mean follicle counts in both categories were generally much higher than counts determined sufficient for detecting CWD in RAMALT tissues [45]. Antemortem data combined across laboratories identified up to 83% of all infected animals; however, it is impractical to consider multi-laboratory testing on a broader scale – for general diagnostic testing, for example. Previous studies have considered testing samples in duplicate experiments to maximize sensitivity [17,18,62], while the present study demonstrated that randomly pairing data from two laboratories resulted in sensitivities which were more similar to that of all laboratories combined. Additional work to assess both optimal sample dilutions and number of experimental repeats would be necessary for further implementation of the RT-QuIC assay for CWD in a diagnostic setting.

Overall sensitivities from individual laboratories, considering both IHC and RT-QuIC, are similar to previous estimates of RAMALT sensitivity in a blinded study conducted on nearly 400 whitetail deer with nearly 80% post-mortem prevalence [18]. Reported sensitivity in that study was 44% for IHC and 68% for RT-QuIC. Previous evaluation of antemortem RAMALT testing in elk placed both IHC sensitivity and RT-QuIC testing at approximately 77% [17]. As multiple factors may influence diagnostic test sensitivity in an infected herd [44,63], future studies on experimental prion amplification techniques should continue to be performed independently and blinded both to and from conventional testing [2].

The rapid, accurate, and reliable diagnosis of prion diseases in cervids is becoming increasingly important as CWD continues to expand outside of its present endemic areas, with new foci discovered on a near-annual basis [51]. Successful management of this disease demands effective surveillance, especially in farmed cervids where sampling is most convenient and practical given animal movement concerns. Cross-validation of the RT-QuIC assay across multiple laboratories in the United States and Canada supports the premise that it can be an effective tool in the antemortem detection of CWD-infected animals, and with additional testing and validation may also prove useful for post-mortem surveillance. The present major limitation is a lack of standardized testing reagents, a factor long since forgotten with more common diagnostic approaches including PCR, ELISA, and IHC. As CWD continues to expand and amplification assays become more widely implemented, it will be important to address this critical limitation.
